# Galectin-9 promotes natural killer cells activity *via* interaction with CD44

**DOI:** 10.3389/fimmu.2023.1131379

**Published:** 2023-03-16

**Authors:** Amirhossein Rahmati, Steven Bigam, Shokrollah Elahi

**Affiliations:** ^1^ School of Dentistry, Division of Foundational Sciences, University of Alberta, Edmonton, AB, Canada; ^2^ Department of Oncology, University of Alberta, Edmonton, AB, Canada; ^3^ Li Ka Shing Institute of Virology, Faculty of Medicine and Dentistry, University of Alberta, Edmonton, AB, Canada

**Keywords:** galectin-9, cancer, COVID-19, cytotoxic effector molecules, cytokines

## Abstract

Natural killer (NK) cells are a potent innate source of cytokines and cytoplasmic granules. Their effector functions are tightly synchronized by the balance between the stimulatory and inhibitory receptors. Here, we quantified the proportion of NK cells and the surface presence of Galectin-9 (Gal-9) from the bone marrow, blood, liver, spleen, and lungs of adult and neonatal mice. We also examined the effector functions of Gal-9^+^NK cells compared with their Gal-9^-^ counterparts. Our results revealed that Gal-9^+^NK cells are more abundant in tissues, in particular, in the liver than in the blood and bone marrow. We found Gal-9 presence was associated with enhanced cytotoxic effector molecules granzyme B (GzmB) and perforin expression. Likewise, Gal-9 expressing NK cells displayed greater IFN-γ and TNF-α expression than their negative counterparts under hemostatic circumstances. Notably, the expansion of Gal-9^+^NK cells in the spleen of mice infected with *E. coli* implies that Gal-9^+^NK cells may provide a protective role against infection. Similarly, we found the expansion of Gal-9^+^NK cells in the spleen and tumor tissues of melanoma B16-F10 mice. Mechanistically, our results revealed the interaction of Gal-9 with CD44 as noted by their co-expression/co-localization. Subsequently, this interaction resulted in enhanced expression of Phospho-LCK, ERK, Akt, MAPK, and mTOR in NK cells. Moreover, we found Gal-9^+^NK cells exhibited an activated phenotype as evidenced by increased CD69, CD25, and Sca-1 but reduced KLRG1 expression. Likewise, we found Gal-9 preferentially interacts with CD44^high^ in human NK cells. Despite this interaction, we noted a dichotomy in terms of effector functions in NK cells from COVID-19 patients. We observed that the presence of Gal-9 on NK cells resulted in a greater IFN-γ expression without any changes in cytolytic molecule expression in these patients. These observations suggest differences in Gal-9^+^NK cell effector functions between mice and humans that should be considered in different physiological and pathological conditions. Therefore, our results highlight the important role of Gal-9 *via* CD44 in NK cell activation, which suggests Gal-9 is a potential new avenue for the development of therapeutic approaches to modulate NK cell effector functions.

## Introduction

Natural killer (NK) cells play an essential role as part of the innate immune system against virus-infected and tumor cells (1). Their deficiency or impaired functions have been linked to enhanced susceptibility to infection and tumor progression (2). Recent evidence has shed light on the adaptive characteristics of NK cells with a memory-like phenotype (3), enforcing an even greater role for these cells in chronic conditions. NK cells are identified as CD3^-^NK1.1^+^ cells in C57BL/6 mice but CD3^-^CD49b^+^ in BALB/c mice. In humans, NK cells are mainly identified as CD3^-^CD16^+^CD56^+^, CD3^-^CD16^+^CD56^-^, and CD16^-^CD56^+^ subsets (4). Consistent with their role in innate immunity, NK cells are widespread throughout the body and can be found in the peripheral blood, liver, lung, spleen, gut, and other places under physiological conditions (2). However, their frequency varies in different tissues and can be altered following pathological conditions.

NK cell effector functions are tightly regulated and depend on the signal received from either stimulatory or inhibitory receptors. As a result, the signal from stimulatory receptors skews the balance toward NK cell activation and vice versa (2). For example, pathological conditions may alter the expression of activating and inhibitory killer-cell immunoglobulin-like receptors (KIRs); lower expression of activating and higher expression of inhibitory KIRs results in NK cell dysfunction (5). Also, it is reported that activated NK cells can be distinguished by surface markers such as CD69, Sca-1(also known as Ly-6A/E), CD25, and KLRG1 (6).

Apart from inhibitory KIRs, NK cells may express other co-inhibitory receptors that are associated with NK cell-impaired effector functions such as PD-1, TIGIT, and TIM-3 (4). Although the role of these co-inhibitory receptors in T cell impairment in the context of chronic infections and cancer has been well-studied (7), their role in NK cell functions has not been free of debate (8). For example, PD-1^+^NK cells exert an impaired degranulation capacity and IFN-γ expression (9). On contrary, TIGIT^+^NK cells display greater cytolytic molecules but lower IFN-γ expression in HIV-infected individuals and cancer patients (4, 10). The role of TIM-3 in NK cell effector functions is highly controversial. One group has documented that TIM-3-expressing NK cells are fully functional in terms of cytokine production and cytotoxicity (11). Other groups have reported TIM-3 as an NK cell exhaustion marker with poor prognosis in patients with cancer (12, 13). Also, it is reported that the interaction of TIM-3 with its ligand Galectin-9 (Gal-9) induces IFN-γ expression in NK cells (14). Given the non-selective binding of TIM-3 and binding to multiple ligands, the different effects of TIM-3 expressing NK cells are suggested to be dependent on the density and type of ligands interacting with (15).

Gal-9 is a β-galactoside-binding lectin that is ubiquitously expressed in immune and non-immune cells (16). This carbohydrate-binding protein is reported to interact with different receptors such as the protein disulfide isomerase (PDI), TIM-3, CD44, CD137, PD-1, and IgE (16–19). Initially identified as an eosinophil chemoattractant, Gal-9 has a wide range of immunomodulatory properties and is involved in cell adhesion, migration, and apoptosis (16, 20). Gal-9 is secreted non-classically as it does not contain an endoplasmic reticulum (ER) signaling sequence (20, 21).

The interaction of Gal-9 with TIM-3 induces an inhibitory signal and is associated with impaired CD8^+^ T cells effector functions (22) Moreover, it has been reported that Gal-9^+^ T cells are terminally exhausted in the peripheral blood of HIV-infected individuals and virus-associated solid tumor patients (10, 19). However, the role of Gal-9 in NK cells has been the subject of controversy. It has been reported that the recombinant (r) rGal-9 upon interaction with TIM-3 enhances cytokine production and degranulation capacity of primed NK cells (14). In contrast, another study suggested that rGal-9 impairs NK cell effector functions *via* downregulating multiple genes associated with cell-mediated cytotoxicity (23). Recently, we reported that rGal-9 enhances cytokine production by NK cells and contributes to the cytokine storm in COVID-19 patients (24, 25). Moreover, we have reported that Gal-9-expressing NK cells display impaired cytolytic molecules expression but increased IFN-γ expression dichotomous to TIGIT-expressing NK cells in HIV and cancer patients (4, 10). These controversial observations enforce further investigation to better understand the role of Gal-9^+^NK cells in different physiological and pathological conditions. In this report, we analyzed the frequency of NK cells in regard to the expression of Gal-9 in different niches in adult and neonatal mice (e.g. spleen, liver, lung, bone marrow, and peripheral blood). Next, we assessed the effector functions of Gal-9^+^ versus their negative counterparts in terms of cytokine, cytolytic molecules expression, and degranulation capacity. Moreover, we identified CD44 as the prime partner of Gal-9 in NK cells in both mice and humans. We also investigated the downstream signaling pathway associated with CD44 in Gal-9^+^NK cells. To gain a deeper insight into the frequency of Gal-9^+^NK cells in pathological conditions, we quantified the percentages of Gal-9^+^NK cells in the spleen of infected mice with *E. coli*. Similarly, we analyzed the proportion of Gal-9^+^NK cells in the spleen and tumor tissues of a melanoma model (B16-F10). Finally, we assessed the functionality of Gal-9^+^NK cells in COVID-19 patients. Our results provide a novel insight into the role of Gal-9 in NK cell function in mice and humans.

## Materials and methods

### Ethics statement

All animal experiments were performed in strict accordance with the recommendations in the Guide for the Care and Use of Laboratory Animals of the Canadian Council for Animal Care. This study was approved by the animal ethics board at the University of Alberta (Protocol # AUP00001021 and AUP00002737).

### Animals

Male and female BALB/c mice were purchased from the Charles River Institute. All animals were maintained and bred under pathogen-free conditions within the animal care facility at the University of Alberta. For the infection-related studies, adult mice were infected with *E. coli* (1 x10^6^ CFU) intraperitoneally (i.p.) as we have described elsewhere (26, 27).

### Animal cancer model

B16-F10 melanoma tumor cells (1×10^5^), originally obtained from the ATCC, were injected subcutaneously on the left flank of mice under anesthesia conditions. Palpable tumors formed 5-7 days after injection and tissues were harvested 16 days after tumor inoculation.

### Human studies

Fresh blood was collected from human subjects infected with SARS-CoV-2 and the peripheral blood mononuclear cells (PBMCs) were isolated by gradient separation using Ficoll-Paque Premium (GE Healthcare, Chicago, USA) according to routine protocols (28, 29). The research ethics boards at the University of Alberta approved blood collection from human subjects with protocol # Pro00099502. Written informed consent was obtained from all the participants in this study.

### Sample processing and cell isolation

Spleens from adult and neonate BALB/c or adult C57BL/c mice were collected. Single-cell suspensions were obtained by grinding tissue between sterile frosted glass slides in 7 mL of 1x RBC lysis buffer and filtering through a 40 µm cell strainer. Splenocytes were resuspended in RPMI with L-glutamine and sodium bicarbonate (Sigma-Aldrich) supplemented with 10% fetal bovine serum (Sigma-Aldrich) and 1% penicillin/streptomycin (Sigma-Aldrich) as we have described previously (30). Similarly, lung and liver samples were ground and resuspended in a culture medium and tissue-residing immune cells were isolated by gradient separation using Ficoll-Paque Premium (GE Healthcare, Chicago, USA). Blood samples from adult BALB/c mice were also subjected to Ficoll-Paque Premium gradient separation. Tumor tissues were dissected aseptically, washed with Hanks’ Balanced Salt Solution two times, cut in small pieces in lysis buffer (DNase [20 U] and Collagenase type IV [2000 U]). Tumor samples were then transferred to 15 ml conical tubes and incubated for 25 mins at 37° C inside a shaking incubator. Then tumor samples were washed, filtered, centrifuged on Ficoll^®^-Paque PREMIUM 1.084.

Isolated cells from the spleen, lung, liver, bone marrow, blood, and tumor tissues were subjected to further analysis. In some experiments, the recombinant human Galectin-9 (rGal-9, 0.25 μg/mL) (Gal Pharma) was added to NK cells in the presence/absence of 100 μg/mL of the anti-CD44 blocking antibody (BD Biosciences, clone KM114) for an hr, as we have reported elsewhere (31). Moreover, in other experiments, the rGal-9 at the same concentration was added to mice splenocytes for an hr before analysis the interaction of rGal-9 with CD71^+^ erythroid cells.

### Antibodies and flow cytometry analysis

Antibodies with specificity to mouse cell surface antigens and cytokines were purchased mainly from BD Biosciences and ThermoFisher Scientific. The following antibodies were used for mice: anti-CD49b [DX5], anti-NK 1.1 (PK136), anti-Gal-9 [RG9-35], anti-IFN-γ [XMG1.2], anti-Perforin [eBioOMAK-D], anti-Granzyme B [NGZB], anti-TNF-α [MP6-XT22], anti-CD107a [1D4B], anti-CD25 (PC61), anti-CD69 (H1-2F3), anti-KLRG-1(2F1), anti-Ly-6A/E also known as SCa-1 (E13-161.7), anti-CD71 (R17217), anti-TER-119 (TER-119), anti-Tim-3 [5D12], anti-CD44 [IM7], anti-CD11b [M1/70], anti-B220 [RA3-6B2], anti-p-LCK [SRRCHA], anti-ERK [20A], anti-AKT [M89-61], anti-MAPK [36/p38 (pT180/pY182)], anti-mTOR [O21-404]. The following antibodies were used for human samples: anti-CD3 [HIT3a], anti-CD16 [B73.1], anti-CD56 [B159], anti-Gal-9 [9M1-3], anti-IFN-γ [45.B3], anti-GzmB [GB11], anti-perforin [dG9], and anti-CD44 [G44-26]. A LIVE/DEAD Fixable Aqua Dead cell stain kit (Thermo Fisher Scientific) was used to exclude dead cells. For intracellular markers, cells were fixed and permeabilized with Cytofix/Cytoperm (BD Biosciences) followed by intracellular staining with the appropriate antibody per our protocols (32). Stained cells (1 x 10^6^) were fixed in 4% paraformaldehyde. Data were acquired on an LSRFortessa Cell Analyzer (BD Biosciences) or Cytek Aurora and analyzed using FlowJo version 10.8 software.

### ImageStream analysis

Surface-stained mouse splenocytes (1 x 10^6^) were fixed in 4% paraformaldehyde. An Amnis ImageStream Mark II (EMD Millipore, ON, Canada) was used to collect at least 10,000 images for each sample as we have previously described (31). Data were analyzed using the IDEAS analysis software package to select single-cell images that were in focus. Next, we performed gating for NK cells (CD49b^+^) followed by gating for cells that co-express Gal-9 and CD44. Colocalization of Gal-9 and CD44 was determined using the IDEAS colocalization wizard.

### NK cell cytotoxicity assay

MHC-I negative target cells (RMA-s cells, kindly provided by Dr. Kevin Kane, University of Alberta) were labeled with CFSE dye and then cultured at ratio of 25:1 (splenocyte: target cell) either total splenocytes with Gal-9^-^NK cells or with cells isolated from liver Gal-9^+^NK cells from BALB/c mice. In brief, the same number of spleen or liver cells were cultured to mimic the physiological condition with target cells. After 4 hr incubation the frequency of killed cells were assessed by flow cytometry as described elsewhere (33).

### Statistical analysis

Statistical comparisons between various groups were performed by a nonparametric student’s t-test Mann-Whitney U test or Kruskal Wallis test for multiple comparisons was used using the Graphpad Prism software (version 9). Results are presented as mean ± standard error of the mean (SEM). *P* < 0.05 was considered statistically significant.

## Results

### Gal-9^+^NK cells are present in the blood circulation and different tissues of adult and neonatal mice

**Figure 1:**
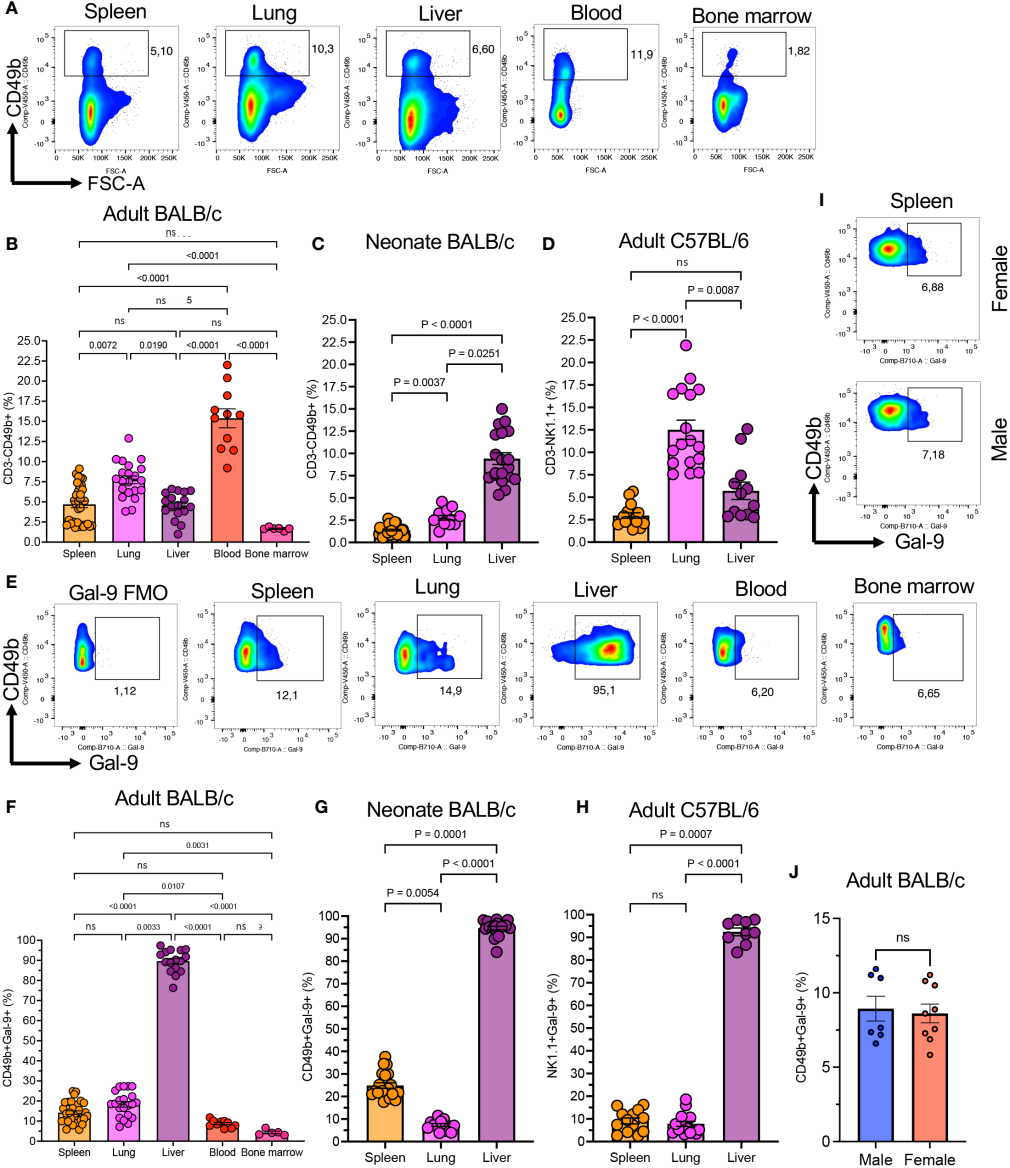
The presence of Gal-9^+^NK cells in different compartments in mice. **(A)** Representative flow cytometry plots showing percentages of total and Gal-9^+^NK cells in the spleen, lung, liver, blood, and bone marrow of adult BALB/c mice. **(B)** Cumulative data of the percentages of NK cells in indicated compartments in adult BALB/c, **(C)** neonatal (day 7-old) BALB/c, and **(D)** adult C57BL/6 mice. **(E)** Representative flow cytometry data of percent Gal-9^+^NK cells in different compartments. **(F)** Cumulative data of percentages of Gal-9^+^NK cells in indicated compartments in adult BALB/c, **(G)** neonatal BALB/c, and **(H)** adult C57BL/6 mice. **(I)** Representative flow plots, and **(J)** cumulative data of percentages of Gal-9^+^NK cells in the spleen of male and female BALB/c mice. Each dot represents data from an animal, mean ± SEM from multiple independent experiments. Fluorescence minus one (FMO), not significant (ns).

We have already reported that Gal-9^+^NK cells have a distinctive phenotype compared to their negative counterparts in the peripheral blood of human subjects (4). Therefore, we decided to investigate the presence/function of Gal-9^+^NK cells in mice. First, we quantified the proportion of NK cells in different compartments in adult (8-10 weeks old) and neonatal BALB/c mice. We found that the frequency of NK cells was significantly higher in the peripheral blood followed by the lung in adult BALB/c mice (**Figures 1A, B**, and **Supplementary Figure 1A**). Moreover, we quantified the percentages of NK cells in the spleen, lung, and liver of neonatal BALB/c mice (7-day old), which showed a different pattern compared to adult mice. We found that the liver was significantly enriched with NK cells compared to the lung/spleen in neonatal BALB/c mice (**Figure 1C**). However, it was impractical to collect blood and bone marrow from pups, and as such we were unable to quantify the frequency of NK cells in the blood circulation and bone marrow of neonatal mice. A similar pattern for the proportion of NK cells in the spleen, lung, and liver of adult C57BL/6 mice was observed when using the NK1.1 marker (**Figure 1D**). However, the percentages of NK cells in the lung of adult BALB/c (average ~7.5%) appeared to be much lower than their counterparts in C57BL/6 mice (~12.5%) **Figures 1B, D**. To ensure that the same population of NK cells being evaluated in both mouse strains, we assessed NK cell frequency in C57BL/6 mice based on the co-expression of NK1.1 and CD49b markers (**Supplementary Figures 1B, C**). However, these observations confirmed our previous conclusions that C57BL/6 mice possess a higher proportion of NK cells in their lungs.

When the expression of Gal-9 on NK cells was analyzed, we observed that a subpopulation of NK cells under normal physiological circumstances express Gal-9 in different tissues in adult BALB/c mice (**Figure 1E**). The frequency of Gal-9^+^NK cells was significantly more pronounced in the liver compared to the spleen, blood, and, bone marrow and lung tissues of adult BALB/c mice (**Figure 1F**). Although the proportion of Gal-9^+^NK cells was similar between the blood and bone marrow, they were more abundant in the spleen and lungs of adult mice (**Figure 1F**). We also quantified the frequency of Gal-9^+^NK cells in neonatal mice (Day-7 old), which showed the liver as the main niche for these cells (**Figure 1G**). However, these observations confirmed that the lung had the least proportion of Gal-9^+^NK cells compared to the spleen and liver in neonatal mice (**Figure 1G**). In consistent with our observations in adult BALB/c mice, we found that the Gal-9^+^NK cells were significantly higher in the liver compared with the spleen/lung of adult C57BL/6 mice (**Figure 1H**). We also compared the frequency of Gal-9^+^NK cells in different tissues between male and female mice but we did not find any difference either in their frequency (**Figures 1I, J**) or the intensity of Gal-9 expression (**Supplementary Figures 1D, E**).

Since the frequency of Gal-9^+^NK cells was lower in the blood and bone marrow our further studies were performed on NK cells in the spleen, liver, and lungs. Taken together, these observations confirm the presence of a substantial number of Gal-9^+^NK cells in different compartments under normal circumstances in both adult and neonatal mice.

### Gal-9^+^NK cells display an activated phenotype compared to their negative counterparts

**Figure 2:**
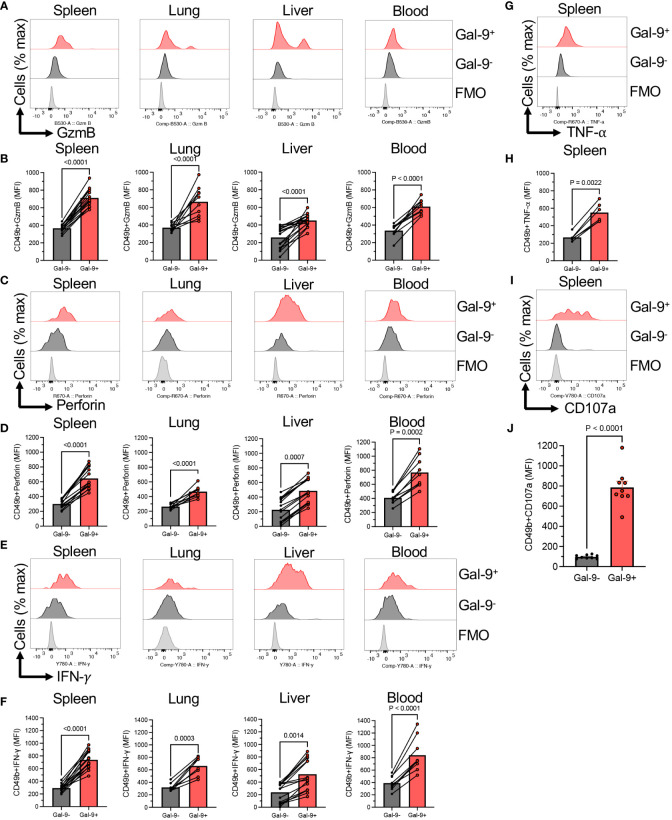
Gal-9^+^NK cells display an activated phenotype compared to their negative counterparts. **(A)** Representative histogram plots, and **(B)** cumulative data showing the intensity of granzyme-B (GzmB) expression as measured by the mean fluorescence intensity (MFI) in Gal-9^+^ and Gal-9^-^NK cells from the spleen, lung, liver and blood of adult BALB/c mice. **(C)** Representative histogram plots, and **(D)** cumulative data showing the intensity of perforin expression as measured by the MFI in Gal-9^+^ and Gal-9^-^NK cells from the spleen, lung, liver and blood of adult BALB/c mice. **(E)** Representative histogram plots, and **(F)** cumulative data showing the intensity of IFN-γ expression as measured by the MFI in Gal-9^+^ and Gal-9^-^NK cells from the spleen, lung, liver and blood of adult BALB/c mice. **(G)** Representative histogram plots, and **(H)** cumulative data showing the intensity of TNF-α expression as measured by the MFI in Gal-9^+^ and Gal-9^-^NK cells from the spleen of adult BALB/c mice. **(I)** Representative histogram plots, and **(J)** cumulative data showing the intensity of CD107a expression as measured by the MFI in Gal-9^+^ and Gal-9^-^NK cells from the spleen of adult BALB/c mice. Each dot represents data from an animal, mean ± SEM from multiple independent experiments. Fluorescence minus one (FMO).

To better characterize the effector functions of Gal-9^+^ versus Gal-9^-^NK cells, we subjected them to further analysis. We found that Gal-9^+^NK cells express significantly a greater level of Granzyme B (GzmB) and perforin expression compared to their negative counterparts in all studied tissues and even in the blood (**Figures 2A–D**). Likewise, Gal-9^+^NK cells had significantly a higher intensity of IFN-γ expression compared with Gal-9^-^NK cells in the spleen, lung, liver, and blood of adult mice (**Figures 2E, F**). Also, we assessed the expression of TNF-α in NK cells, which confirmed a similar trend as IFN-γ expression in Gal-9^+^NK cells (**Figures 2G, H**). Considering the role of Gal-9 in immune cell effector function impairments (19), We speculated that a higher cytokine and cytolytic molecules content in Gal-9^+^NK cells might be associated with a lower degranulation capability. Surprisingly, we found that Gal-9^+^NK cells displayed a more robust degranulation capacity compared to their negative counterparts (**Figures 2I, J**). Collectively, our observations imply that Gal-9^+^NK cells have a more activated phenotype regardless of their niche, animal strain, and animal age.

### Gal-9 preferentially interacts with CD44 on NK cells

**Figure 3:**
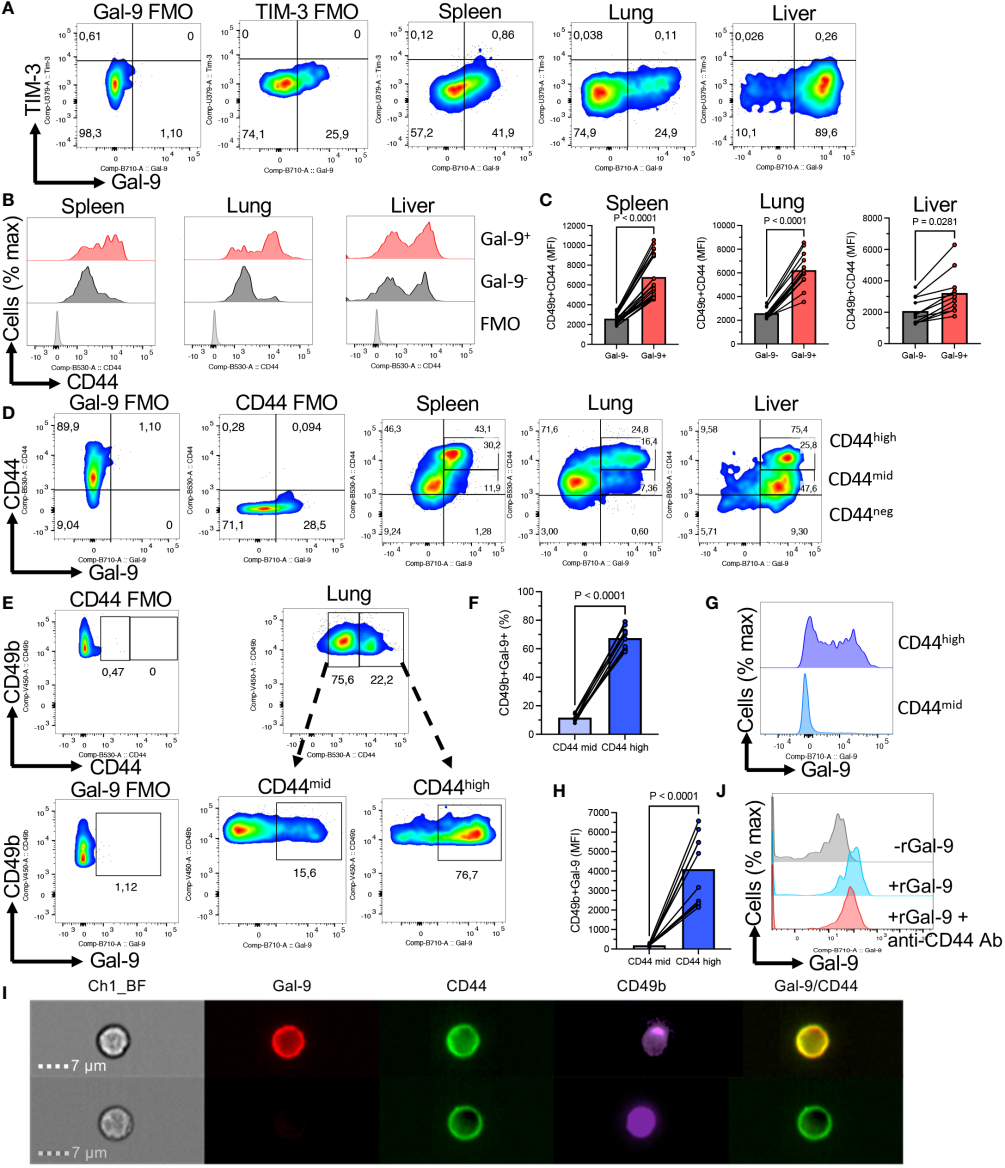
Gal-9 is preferentially co-expressed with CD44^high^ NK cells. **(A)** Representative flow cytometry plots of the expression of TIM-3 in NK cells from the spleen, lung, and liver of adult BALB/c mice. **(B)** Representative histogram plots, and **(C)** cumulative data showing the intensity of CD44 expression as measured by the MFI in Gal-9^+^ and Gal-9^-^NK cells from the spleen, lung, and liver of adult BALB/c mice. **(D)** Representative flow cytometry plots showing CD44 and Gal-9 co-expression in NK cells from the spleen, lung, and liver of adult BALB/c mice. **(E)** Representative flow cytometry plots, and **(F)** cumulative data illustrating percentages of Gal-9^+^NK cells in CD44^high^ and CD44^mid^ subpopulation of NK cells in the lung. **(G)** Representative histogram plots, and **(H)** cumulative data illustrating the intensity of Gal-9 expression in CD44^high^ and CD44^mid^ subpopulation of NK cells in the lung. **(I)** Representative Image Stream plots showing Gal-9 and CD44 co-localization on a CD49b cell (NK cell). **(J)** Histogram plots of Gal-9 expression in NK cells from a spleen of BALB/c mouse in the absence or presence of rGal-9 (0.25 μg/ml) and/or anti-CD44 antibody. Each dot represents data from an animal, mean ± SEM from multiple independent experiments. Fluorescence minus one (FMO). Chanel 1 bright field (Ch1-BF). The subdivision gates in Figure 3D cover some of the cells between gates and as result, the quadrant total for the liver is higher (75.4%) than the sum of 25.8% + 47.6% (73.4%).

To gain insight into the underlying mechanism of hyperimmune activation in Gal-9^+^NK cells, we conducted further investigations. It is reported that Gal-9 binds/interacts with different receptors (e.g. TIM-3, CD45, CD44, Dectin-1, etc.) (16, 34–36). Therefore, we quantified the expression of TIM-3 on Gal-9^+^/Gal-9^-^NK cells in different tissues of adult mice. Interestingly, we found negligible expression of TIM-3 on NK cells regardless of their niche (**Figure 3A**). As such, we did not observe any Gal-9/TIM-3 co-expression on NK cells under homeostatic conditions in mice (**Figure 3A**). These observations precluded the interaction of Gal-9 with TIM-3.

**Figure 4:**
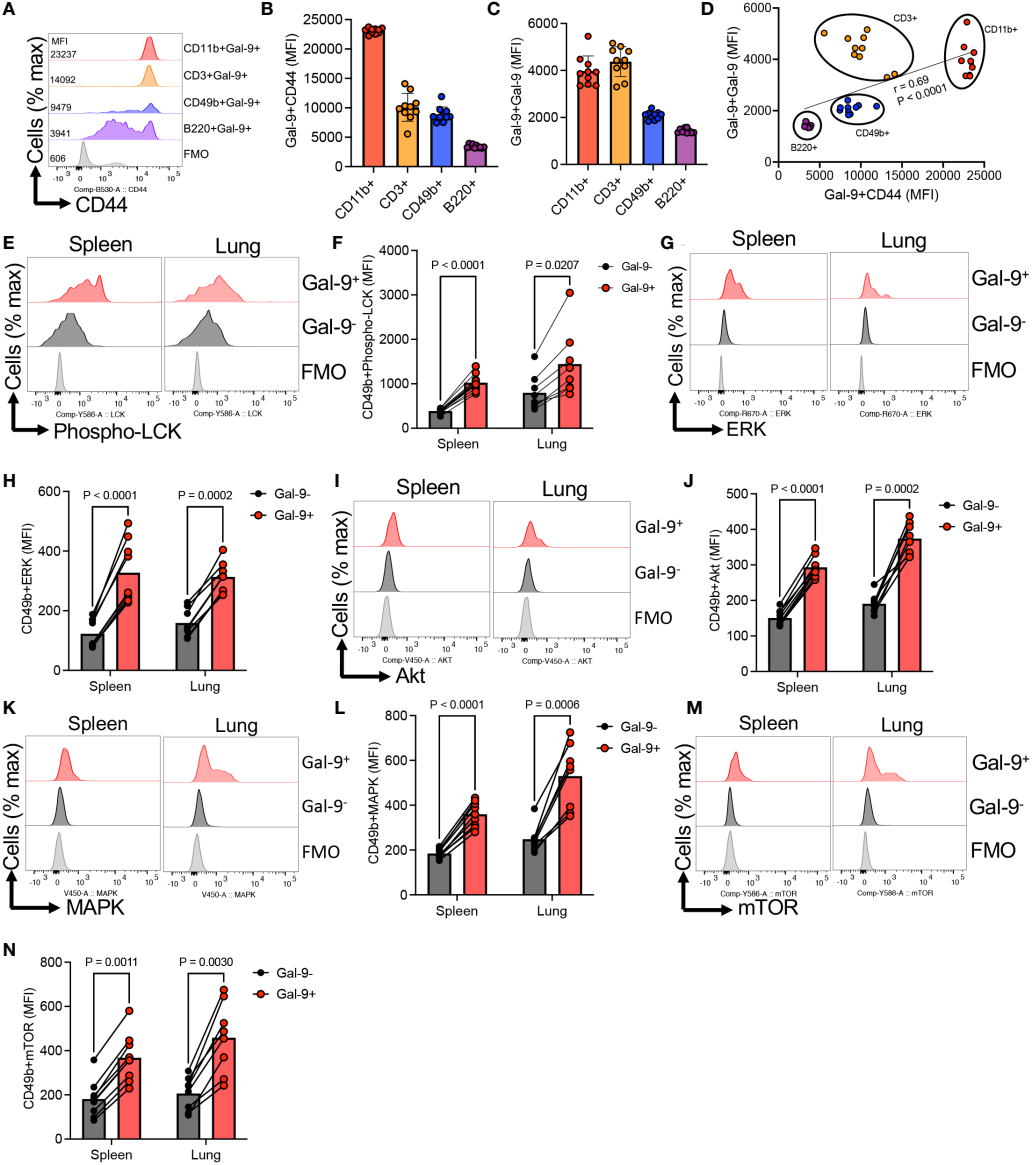
Gal-9 interaction with CD44 results induces NK cell activation. **(A)** Representative histogram, and **(B)** cumulative data of the mean fluorescence intensity (MFI) of CD44 expression in CD11b, CD49b, B220, and CD3 T cells from the spleen of adult BALB/c mice. **(C)** Cumulative data showing the MFI of Gal-9 expression in Gal-9^+^CD11b, CD49b, B220, and CD3 T cells from the spleen of adult BALB/c mice. **(D)** Showing the correlation between the intensity of Gal-9 with the intensity of CD44 expression in different indicated immune cells from the spleen of adult BALB/c mice. **(E)** Representative histogram plots, and **(F)** cumulative data of the intensity of Phospho-LCK expression in Gal-9^+^ versus Gal-9^-^NK cells in the spleen and lung of adult BALB/c mice. **(G)** Representative histogram plots, and **(H)** cumulative data of the intensity of ERK expression in Gal-9^+^ versus Gal-9^-^NK cells in the spleen and lung of adult BALB/c mice. **(I)** Representative histogram plots, and **(J)** cumulative data of the intensity of Akt expression in Gal-9^+^ versus Gal-9^-^NK cells in the spleen and lung of adult BALB/c mice. **(K)** Representative histogram plots, and **(L)** cumulative data of the intensity of MAPK expression in Gal-9^+^ versus Gal-9^-^NK cells in the spleen and lung of adult BALB/c mice. **(M)** Representative histogram plots, and **(N)** cumulative data of the intensity of mTOR expression in Gal-9^+^ versus Gal-9^-^NK cells in the spleen and lung of adult BALB/c mice. Each dot represents data from an animal, mean ± SEM from multiple independent experiments. Infection study (O&P) from two independent experiment. Fluorescence minus one (FMO).

Our further studies suggested that CD44 might be the main partner of Gal-9 on NK cells. This was illustrated by a more pronounced expression of CD44 in Gal-9^+^NK cells compared to their Gal-9^-^ counterparts (**Figures 3B, C**). We further analyzed the co-expression of Gal-9 with CD44. First of all, we noted that NK cells can be divided based on the expression of CD44 into CD44^high^ and CD44^mid/low^ NK cells (**Figure 3D**). When the co-expression of Gal-9 was further assessed, we found that Gal-9 was preferentially co-expressed with CD44^high^ NK cells in the spleen and lung (**Figure 3D**). However, this was not the case for the liver NK cells as the majority of them express Gal-9 (**Figure 3D**). To better clarify this finding, we compared the percentages of Gal-9 expressing NK cells in CD44^high^ and CD44^mid/low^ cells. These analyses confirmed that Gal-9 was strongly associated with the CD44^high^ NK subset as illustrated by the significant enrichment of Gal-9^+^NK cells in the CD44^high^ subset of NK cells (**Figures 3E, F**). Furthermore, we quantified the intensity of Gal-9 expression in CD44^high^ and CD44^mid/low^ NK cells in the lung, which confirmed a greater intensity of Gal-9 expression in CD44^high^ compared with CD44^mid/low^ NK cells (**Figures 3G, H**). To better visualize the co-expression of CD44 with Gal-9 on NK cells, we performed image stream analysis according to our protocols (37, 38). These observations indicated that Gal-9 is col-localized with CD44 on NK cells (**Figure 3I**, and **Supplementary Figure 1F**). To further assess the interaction of Gal-9 and CD44, we treated NK cells with the anti-CD44 antibody (an hr) followed by treatment with the rGal-9 (0.25 μg for an hr). We observed that CD44 blockade abrogates the binding of rGal-9 to NK cells *in vitro* (**Figure 3J**). Alternatively, we used CD71^+^ erythroid cells (CECs) (39, 40), as proof of concept, considering that these cells have negligible surface Gal-9 and a small portion of them express CD44. We found that treatment of these cells with rGal-9 resulted in an increased proportion of Gal-9^+^CD44^+^ cells without any changes in the CD44^-^CEC subset (**Supplementary Figure 2**). It is worth mentioning that treatment with rGal-9 increased the proportion of CD44 expressing CECs *in vitro* (**Supplementary Figure 2**). This observation suggests that rGal-9 may enhance the expression of CD44, however, further studies are required to determine this possibility. Although these cells are different than NK cells, our observations show the tendency of Gal-9 towards CD44^+^ cells. Since Gal-9 is ubiquitous and interacts with different immune cells, we decided to determine whether CD44 is a common target for Gal-9 in different immune cells in mice. For this, we compared the expression of CD44 in different splenic immune cells in adult mice. We found that CD11b^+^ cells followed by T cells and NK cells had the greatest levels of CD44 expression but B cells had the least (**Figures 4A, B**). In line with this hypothesis, we observed a higher expression of Gal-9 in CD11b^+^ cells and T cells followed by NK cells (**Figure 4C**). This was further confirmed as we found a strong positive correlation between Gal-9 with CD44 expression in different immune cells (**Figure 4D**). Collectively, our results suggest that CD44 is the major partner for Gal-9 in different immune cells, in particular, NK cells.

### Gal-9:CD44 interaction regulates NK cell activation

To gain further mechanistic insight into the Gal-9:CD44 interactions in NK cell function, we analyzed the activation of intracellular signaling pathways known to operate downstream of CD44 (41). In agreement, we found enhanced expression of Phspho-LCK in Gal-9^+^ versus Gal-9^-^NK cells in the spleen and lung of adult mice (**Figures 4E, F**). Similarly, we noted a significant elevation in the expression of ERK in Gal-9^+^ compared to Gal-9^-^ NK cells (**Figures 4G, H**). Likewise, the expression of Akt, MAPK, and mTOR were significantly higher in Gal-9^+^ versus Gal-9^-^ NK cells, respectively (**Figures 4I–N**).

**Figure 5:**
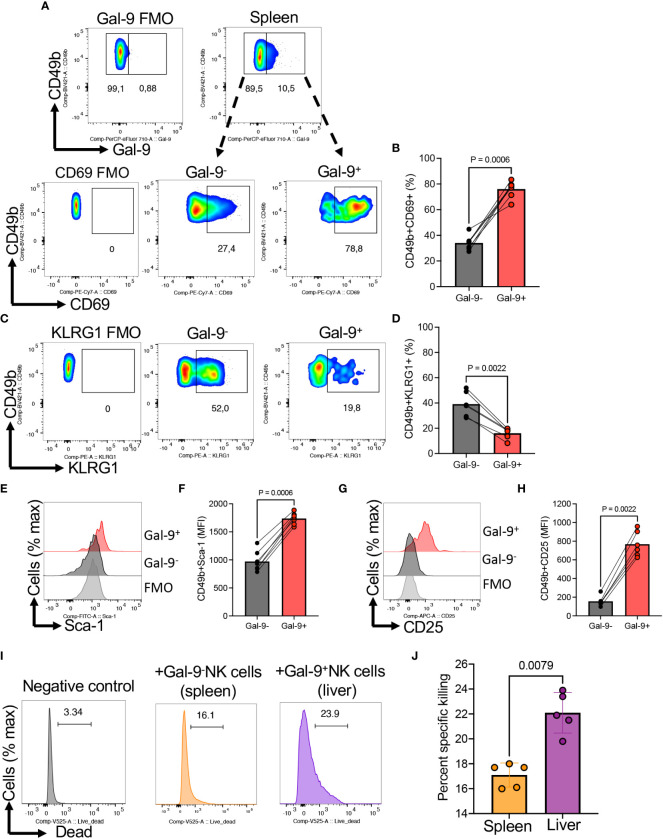
The expression of CD69, KLRG-1, Sca-1, and CD25 in Gal-9^+^NK cells. **(A)** Representative flow cytometry plots, and **(B)** cumulative data of percentages of CD69 expressing NK cells among Gal-9^-^ or Gal-9^+^ NK cells of BALB/c mice. **(C)** Representative flow cytometry plots, and **(D)** cumulative data of percentages of KLRG1 expressing NK cells among Gal-9^-^ or Gal-9^+^ NK cells of BALB/c mice. **(E)** Representative flow cytometry plots, and **(F)** cumulative data of the intensity of Sca-1 expression among Gal-9^-^ or Gal-9^+^ NK cells of BALB/c mice. **(G)** Representative flow cytometry plots, and **(H)** cumulative data of the intensity of CD25 expression among Gal-9^-^ (splenocytes) or Gal-9^+^ (liver cells) NK cells of BALB/c mice**. (I)** Representative plots, and **(J)** cumulative data of percent specific killing of target cells (RMA-s cells) in the presence and absence of Gal-9^+^NK cells in splenocytes of mice. Each dot represents data from an animal, mean ± SEM from multiple independent experiments. The mean fluorescence intensity (MFI). Fluorescence minus one (FMO).

Moreover, we subjected NK cells from the spleen to further investigations for the expression of co-stimulatory/inhibitory markers. We found that Gal-9^+^NK cells were enriched with a higher proportion of CD69 but reduced KLRG-1 expressing NK cells compared to their negative counterparts (**Figures 5A–D**). Given the activation status of Gal-9^+^NK cells, we speculated that these cells may express a higher level of stem cell Ag1 (Sca-1), as a marker of activated NK cell, which plays a role in NK cell response to infection (42). Interestingly, we observed that Gal-9^+^NK cells had significantly a higher level of this activation marker that their negative counterparts (**Figures 5E, F**). Similarly, we observed a greater expression of CD25 in Gal-9^+^ than in Gal-9^-^NK cells (**Figures 5G, H**). To further associate the hyperactivation status of Gal-9^+^NK cells with their cytotoxicity, we performed cytotoxicity assay at 25:1 ratio of spleen/liver cells with target cells (1 x 10^6^: 40,000 RMA-s cells) for 4 hr without any stimulation. We found that liver Gal-9^+^NK cells exhibited a greater specific killing of target cells (**Figures 5I, J**).

Together, these data suggest the relatedness of the CD44 and Gal-9 signaling in inducting NK cell activation.

### Gal-9^+^NK cells are increased in the spleen of mice following infection with *E. coli*

**Figure 6:**
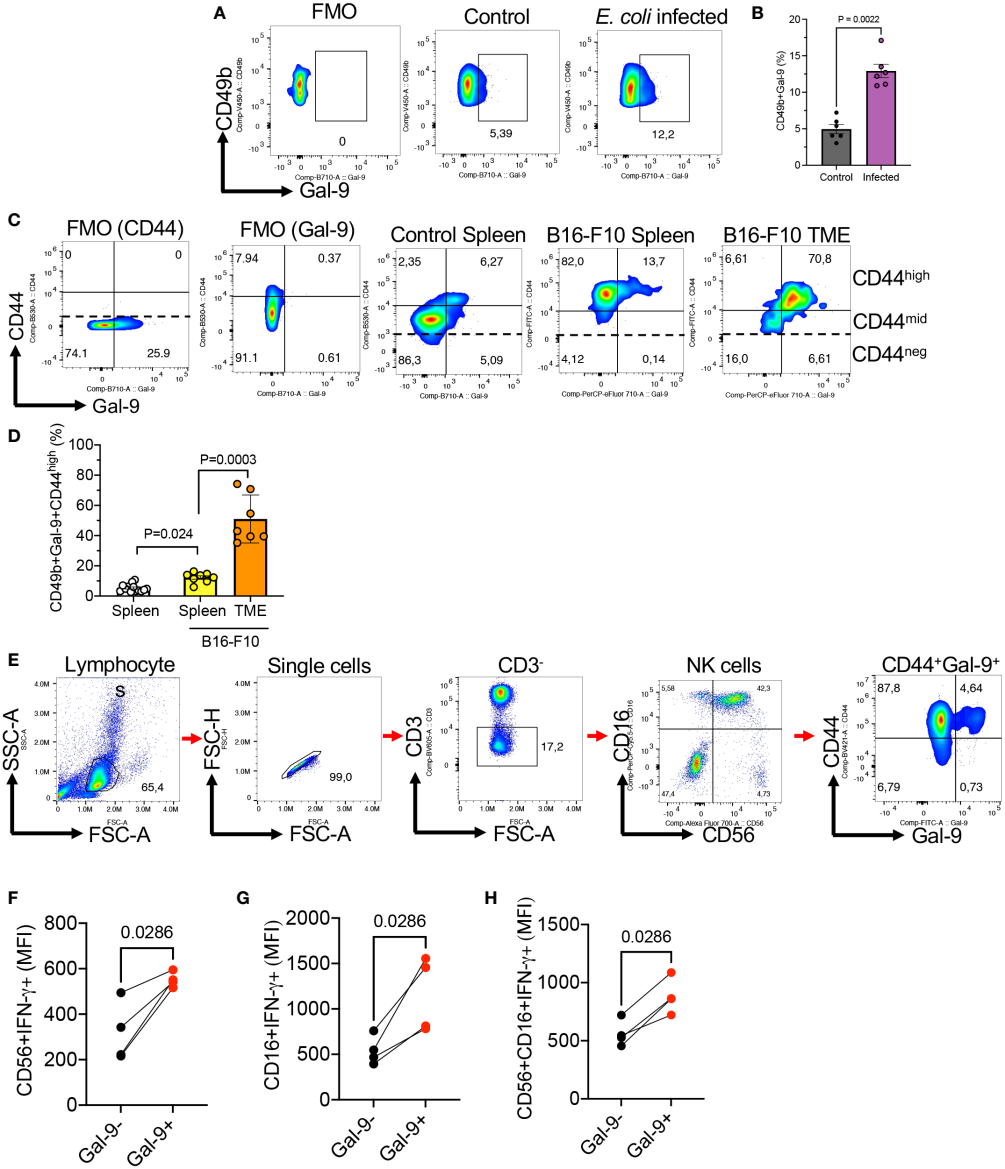
The expansion of Gal-9^+^NK cells in the spleen and tumor tissues of B16-F10 melanoma model and the expansion of Gal-9^+^NK cells in COVID-19 patients. **(A)** Representative flow cytometry plots, and **(B)** cumulative data of the frequency of NK cells in the spleen of control or infected adult mice with *E*. *coli* 48 hr post-infection. Each dot represents data from an animal. **(C)** Representative flow cytometry plots showing the co-expression of CD44 and Gal-9 in NK cells from the spleen of tumor naïve or the spleen and tumor microenvironment (TME) of tumor bearing mice (B16-F10 melanoma). **(D)** Cumulative data of the percentages of Gal-9^+^NK cells in the spleen of tumor naïve or the spleen and TME of tumor-bearing mice. **(E)** Representative gating strategy for human Gal-9^+^NK cells. **(F)** Cumulative data of IFN-γ expression in Gal-9^+^ and Gal-9^-^ NK cells among CD56^+^CD16^-^, **(G)** CD16+CD56-, and **(H)** CD16^+^CD56^+^ subset of human NK cells. Each dot represents data from an animal or human study subject. The mean fluorescence intensity (MFI).

Considering a higher activity of Gal-9^+^NK cells in terms of cytokine and cytolytic molecule expression, we decided to determine the impact of infection on this NK subset in mice. As proof of concept, adult mice were infected with *E. coli* (1 x10^6^ CFU, intraperitoneally). Two days later their splenocytes were subjected to further analysis. These studies showed a significant expansion of Gal-9^+^NK cells in the spleen of infected mice (**Figures 6A, B**). Therefore, our observations suggest that the expansion of Gal-9^+^NK cells might be an indicative of enhanced innate immunity against infection.

### Gal-9^+^NK cells are highly abundant in the tumor microenvironment

To determine the presence of Gal-9^+^NK cells in pathological conditions, we investigated the presence of NK cells in the spleen and tumor tissues of tumor-bearing mice (B16-F10 melanoma). We did not observe any significant difference in the frequency of total NK cells in the spleen of tumor-bearing mice compared to controls. However, we found a significant expansion of Gal-9^+^NK cells in the spleen of tumor-bearing mice versus controls (**Figures 6C, D**). In particular, we observed that the majority of tumor resident NK cells expressed Gal-9 (**Figures 6C, D**). These observations showed that Gal-9 was mainly expressed on CD44^high^ NK cells (**Figure 6C**). Gal-9^+^NK cells in tumor-bearing mice exhibited a more robust effector functions as we reported for their counterparts in healthy mice. These observations support the interaction of Gal-9 with CD44^high^ in pathological conditions. However, further studies are needed to determine the prolonged impact of these interactions on NK cells effector functions.

### Gal-9 interacts with CD44 in human NK cells

We have previously reported that the frequency of Gal-9^+^NK cells get expanded in HIV-infected individuals and patients with virus-associated solid tumors (4, 10). To determine whether our observations in mice were applicable to humans, we investigated the interaction of CD44 with Gal-9 in human NK cells. Since healthy individuals have negligible levels of Gal-9^+^NK cells, we studied the frequency of Gal-9^+^NK cells in a few SARS-CoV-2 infected individuals. These results confirmed our observations in mice that Gal-9 was preferentially co-expressed with CD44^high^ NK cells (**Figure 6E**). Overall, these results suggest that Gal-9 has the propensity of interacting with CD44 on NK cells in both mice and humans. Despite the interaction of Gal-9 with CD44 we found a dichotomy in terms of effector functions in human NK cells. We observed Gal-9^+^NK cells in humans display a greater IFN-γ expression (**Figures 6F–H**) without a significant difference in their cytolytic molecules expression. These observations suggest differences in Gal-9^+^NK cells effector functions between mice and humans in different pathological conditions.

## Discussion

NK cells as primacy innate effectors play an essential role against intracellular pathogens and tumors. Emerging evidence supports a regulatory role for NK cells in minimizing inflammation and enhancing tissue repair (43). In contrast, there are reports on the involvement of these innate immune cells in autoimmune conditions (44). In this report, we found that NK cells were highly abundant in the blood circulation and lungs than the liver, spleen, and bone marrow niches in adult mice. However, in newborns, this pattern was different where we found the liver as the major source of NK cells. This is in line with the predominant innate immunity role of the liver in host defense against pathogens (45). Interestingly, we observed that the liver of either adult or neonatal mice was highly enriched with Gal-9 expressing NK cells. Moreover, the intensity of Gal-9 was exceptionally elevated in the liver NK cells compared with their counterparts in other compartments. This observation suggests an important role for Gal-9 in immune homeostasis in the liver under normal physiological conditions. Given the fact that Gal-9 is highly expressed in the liver (46), it explains why Gal-9^+^NK cells are abundant in the liver compared to other compartments. Considering the role of Gal-9 in the regulation of hepatic inflammation, further studies are warranted to better elucidate the role of Gal-9^+^NK cells under steady circumstances in the liver. Apart from the liver, we observed a substantial proportion of Gal-9^+^NK cells in the spleen, lung, and blood circulation of mice, which suggests a broad role for this NK cell subset under homeostatic conditions.

Functionally, we observed that Gal-9 expression was associated with enhanced cytolytic molecules expression (e.g. GzmB and perforin) and IFN-γ/TNF-α production in NK cells. These observations imply that Gal-9^+^NK cells exhibit more robust effector functions than their negative counterparts. Considering that Gal-9 lacks a transmembrane domain (16), it’s unclear whether Gal-9 get released by other cells and subsequently binds to its receptor on NK cells or it is translocated from the cytosol of NK cells to their membrane. Gal-9 is ubiquitous and can be sourced from different immune, non-immune, and tumor cells, or apoptotic cells (24, 25, 47). Alternatively, Gal-9 presence could be subsequent to disease chronicity and persistent infection (48).

Although there is no report on the presence and function of Gal-9^+^NK cells under normal physiological conditions in different tissues of mice, it is documented that Gal-9 promotes the survival of B16-F10 melanoma-bearing mice through NK cell modulation (49). However, this study ruled out any direct role for Gal-9 in NK cell-enhanced activity. Instead, it provided an indirect role for Gal-9 in promoting NK cell-mediated anti-tumor function *via* the expansion of a subset of plasmacytoid dendritic cell-like phenotype (49). Considering the robust effector functions of Gal-9^+^NK cells and their expansion in the spleen/tumor tissues of B16-F10 melanoma model, we speculate this NK cell subpopulation may play a protective role against the tumor. Despite this evidence, another group has reported an inhibitory role for Gal-9-mediated NK cell activity (23). A possible explanation for the differential effects of Gal-9 on NK cells might be related to the interacting receptor. For example, the interaction of Gal-9 with TIM-3 is reported to be associated with NK cell exhaustion (11). Similarly, the interaction of Gal-9 with TIM-3 was found to inhibit NK cell effector functions in hepatitis B infection (50). This suggests that Gal-9 *via* interaction with TIM-3 may impair NK cell activity. Although the role of TIM-3 in NK cell function is controversial, a couple of studies suggested that TIM-3 contributes to NK cell activity (11, 14). For instance, elevated viral load with concurrent chronic inflammation has been linked to NK cell activation and higher TIM-3 expression in HIV-infected individuals (51). However, in this study, we did not observe any TIM-3^+^NK cells in different compartments of mice. This observation excludes a role for TIM-3 in the enhanced NK cell activity in Gal-9^+^NK cells.

To explain the underlying mechanism of NK cells activity, we hypothesized that greater cytotoxic contents and cytokine expression in Gal-9^+^NK cells might be due to their deficient degranulation capacity rather than having higher granules content. Surprisingly, our further investigations showed that the Gal-9^+^NK subset exhibited a greater degranulation capacity (CD107a expression) than their Gal-9^-^ counterparts. This observation supports a higher activation status for Gal-9^+^NK cell despite indiscriminate and constant degranulation. The hyperactivation status of Gal-9^+^NK cells was further supported when Gal-9^+^NK cells significantly increased target cell specific killing in splenocytes *in vitro*.

Although we have defined Gal-9^+^ as activated NK cells, one might argue that these cells are functionally different to enhance adaptive immunity. Since IFN-γ can upregulate MHC class I, it increases the potential for CD8 T cell recognition of infected target cells (52–54). Alternatively, these activated NK cells may enhance the recruitment of immune cells (e.g. macrophages) to the site of infection as reported in other conditions (55). While a proinflammatory environment associated with enhanced cytokine production is critical for orchestrating a robust innate immune response to mediate pathogens control in the early stage of disease, excessive/prolonged production of cytokines (e.g. TNF-α, IFN-γ) may result in systemic inflammation (56, 57). However, delineating the role of Gal-9^+^NK cells in mice requires further investigation. In contrast to mice, Gal-9^+^NK cells are scarce in the blood circulation of healthy human individuals but they become expanded in the peripheral blood of HIV and cancer patients (4, 10). Functionally, Gal-9^+^NK cells display impaired cytotoxicity but enhanced cytokine production capacity (e.g. IFN-γ) in patients with HIV and cancer (4, 10).

Our observations suggest that higher cytolytic granules in Gal-9^+^NK cells may enhance their ability to eliminate infected target cells since high granule content (e.g. GzmB and perforin) is crucial for NK cell-mediated cytotoxicity (58). Nevertheless, the involvement of Gal-9^+^NK cells, with a higher level of granule contents and cytokine expression, in immunopathology is unclear and merits further investigations. It is worth mentioning that the regulation of NK cells is dependent on an array of activating and inhibitory receptors and there might be other unexplored activating/inhibitory receptors on the Gal-9^+^NK cells that contribute to the observed functional phenotype.

Considering the diverse immunomodulatory roles of Gal-9 in various immune and non-immune cells, our knowledge of underlying signaling events remains limited. Given the fact that our results did not support a definite role for TIM-3 as the potential partner of Gal-9, we conducted further investigations. We observed that Gal-9 interacts with CD44 on NK cells, which may signal the epigenetic imprinting of cytokine genes (e.g. IFN-γ *via* LCK activation), resulting in enhanced NK cell activity as reported for T cells (59, 60). This was illustrated by the enhanced expression of Phospho-LCK, ERK, Akt, MAPK, and mTOR in Gal-9^+^NK cells. This agrees with the well-documented role of CD44 in activating a series of key signaling pathways (e.g. MAPK, PI3K/AKT, etc.) (61). Moreover, we noted a higher expression of activation markers such as CD69, CD25, and Sca-1 but reduced expression of KLRG1, an inhibitory receptor (62), in Gal-9^+^NK cells. Although these observations support an activated phenotype for Gal-9^+^NK cells, this immune activation status cannot be directly attributed to Gal-9/CD44 interactions without further in-depth analysis.

Although CD44 lacks intrinsic kinase activity, it is reported that *via* interaction with cell surface factors initiates subsequent activation of downstream pathways (63). Therefore, it is likely that CD44-signaling transduction takes place *via* Gal-9-mediated CD44 clustering as reported in neutrophils (31). Considering the role of CD44 in immune cell proliferation, adhesion, and migration (31, 64), further studies are needed to determine whether such properties are modulated in Gal-9^+^NK cells.

It is worth mentioning that despite our focus on Gal-9/CD44 interactions in this study, there are some similarities between CD44 and TIM-3 downstream signaling. It is reported that upon T cell activation, TIM-3 is recruited to the immunologic synapse and interacts with LCK (65). Gal-9 interaction with TIM-3 triggers the phosphorylation of Tyr256/263 by the tyrosine kinase IKT, thereby allowing TIM-3 to exert an inhibitory signal (66). Moreover, the interactions of TIM-3 with CD45 and CD148 result in the disruption of the immunological synapse as a mechanism of T cell inhibition (65). In contrast, TIM-3 expression has been linked to enhanced AKT and mTOR signaling, which indicates a sign of co-stimulation (67). TIM-3 is reported to inhibit ERK and NF_k_B p65 signaling but fails to suppress IFN-γ production by NK cells (68), which is also documented for increased IFN-γ expression in NK cells following interaction with the recombinant Gal-9 (14). These observations have generated some controversy regarding the role of TIM-3 as a co-inhibitory or co-stimulatory molecule.

Inconsistent with animal studies, we found the co-expression of Gal-9 with CD44^high^ in human NK cells. It is worth mentioning that Gal-9^+^NK cells are absent or scarce in human blood circulation, however, they get expanded in pathological conditions. Our observations highlight the discrepancy in regards to the presence of Gal-9^+^NK cells under normal physiological conditions in mice versus humans. However, we found the expansion of Gal-9^+^NK cells in the blood circulation of SARS-CoV-2 infected individuals. Despite the interaction of Gal-9 with CD44 in human NK cells, the biological outcomes were different from mice. Although Gal-9^+^NK cells in HIV-infected individuals and cancer patients display a greater IFN-γ expression but lower cytolytic molecules expression, this was different in COVID-19 patients. These observations indicate the complexity of pathological conditions on NK cell effector functions.

We are aware of our study limitations. While it is plausible that Gal-9 and CD44 interaction enhances the activation status of NK cells, functional studies such as Gal-9/CD44 co-immunoprecipitation and genetic knockdown/molecular perturbation studies are needed to conclude that such an interaction exists and that it results in increased cytotoxic potential of NK cells in mice. Another caveat is the inability to investigate the outcomes of Gal-9^+^NK cells depletion in pathological conditions such as chronic inflammation and cancer in animal models. Such studies will provide insight into the role of Gal-9 in NK cell effector functions in chronic conditions. We were unable to determine the presence of Gal-9^+^NK cells in different human tissues due to the lack of access. Therefore, the frequency and functionality of residential Gal-9^+^NK cells in human tissues need to be investigated.

Taken together, our findings revealed an undescribed role for Gal-9 in NK cell activity in homeostatic conditions. More importantly, we observed that Gal-9 expression was associated with enhanced activation of NK cells in terms the expression of cytotoxic mediators, activation markers, cytotoxicity, and IFN-γ/TNF-α expression in mice. In particular, we found the interaction of Gal-9 with CD44 as a possible underlying mechanism associated with enhanced NK cell activity. Therefore, our data provide a novel role for Gal-9 in NK cell functions and may help elucidate how Gal-9 influences the functionality of NK cells in other acute and chronic conditions.

## Data availability statement

The original contributions presented in the study are included in the article/**Supplementary Material**. Further inquiries can be directed to the corresponding author.

## Ethics statement

The studies involving human participants were reviewed and approved by the research ethics boards at the University of Alberta. The patients/participants provided their written informed consent to participate in this study. The animal study was reviewed and approved by the animal ethics board at the University of Alberta.

## Author contributions

AR performed most of the experiments, analyzed the data, designed the figures, and wrote the first draft of the methods section. SB performed some of the experiments, analyzed data, assisted in the figures design, and wrote the first draft of the methods section. SE proposed and conceived the original idea, designed and supervised all of the research, secured resources, provides guidance in data analysis, and wrote the manuscript. All authors contributed to the article and approved the submitted version.

## Supplementary material

Supplementary Figure 1
**(A)** The gating strategy for NK cells and Gal-9^+^NK cells. **(B)** Representative flow cytometry plots, and **(C)** cumulative data of NK cells defined as CD3^-^CD49b^+^NK1.1^+^ cells in the spleen, lung, and liver of C57BL/6 mice. (**D**) Representative histogram plots, and **(E)** cumulative data of the intensity of Gal-9 expression in adult male and female mice. **(F)** Representative gating strategy for the ImageStream analysis. Each dot represents data from an animal, mean ± SEM from multiple independent experiments. Fluorescence minus one (FMO), the mean fluorescence intensity (MFI).

Supplementary Figure 2 The gating strategy for the splenic CD71^+^ erythroid cells (21-day old BALB/c mice) and the co-expression of CD44 and Gal-9 following treatment of these cells with the rGal-9 for an hr (0.25 μg/ml).
