# Supplementary figures and images for: Galectin-9 promotes natural killer cells activity via interaction with CD44
Source: Front Immunol. 2023 Mar 16;14:1131379. doi: 10.3389/fimmu.2023.1131379 (PMC10060867; doi:10.3389/fimmu.2023.1131379)

# Supplemental Figure 1

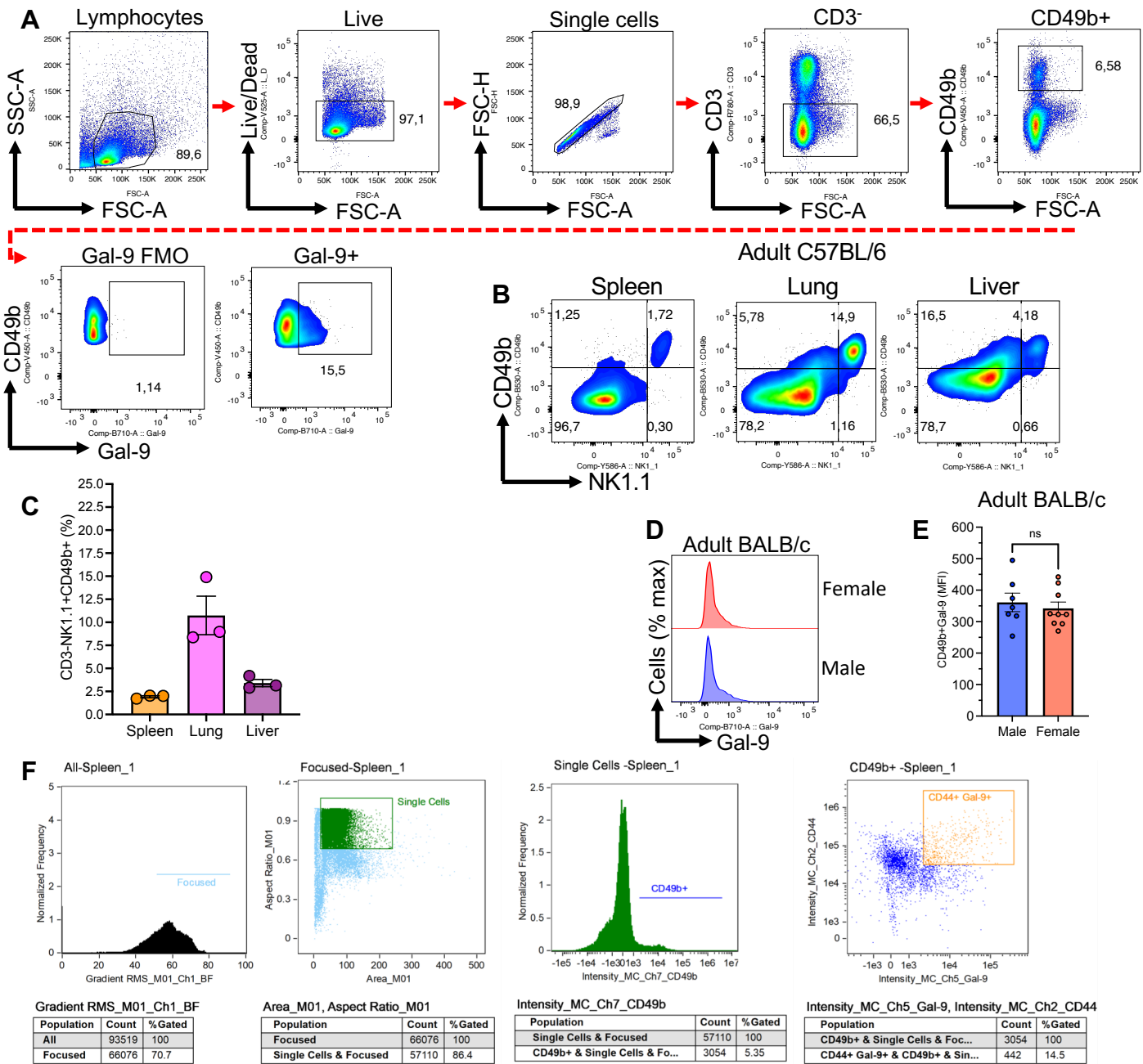

Supplemental Figure 2

C57BL/6 Spleen day 21 male

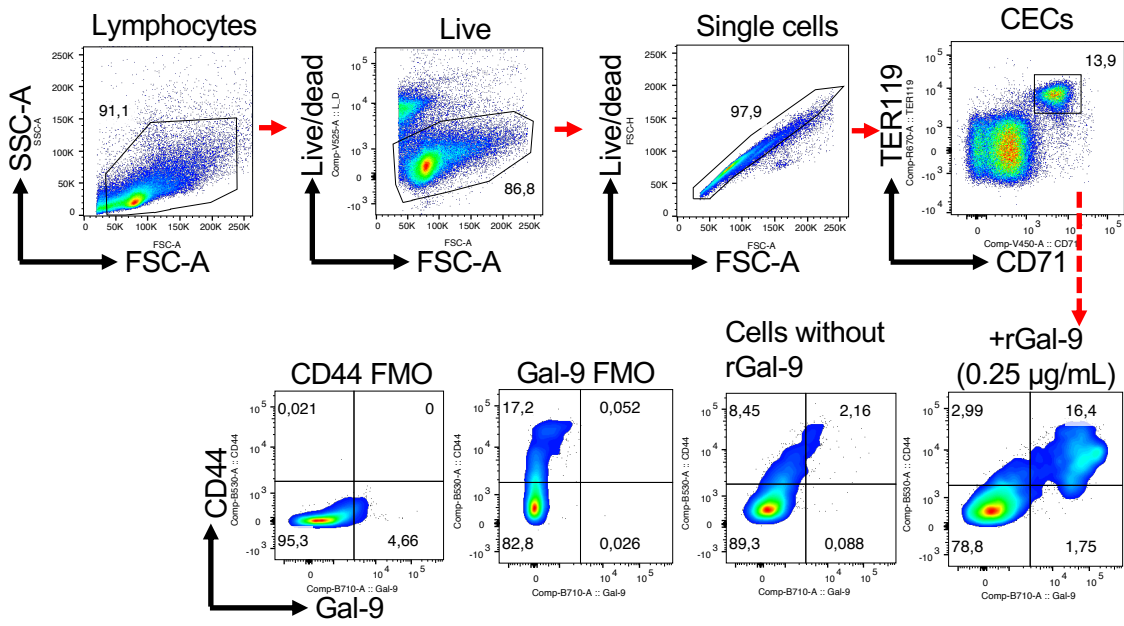

Supplement: Supplementary Figure 1 — (A) The gating strategy for NK cells and Gal-9+NK cells. (B) Representative flow cytometry plots, and (C) cumulative data of NK cells defined as CD3-CD49b+NK1.1+ cells in the spleen, lung, and liver of C57BL/6 mice. (D) Representative histogram plots, and (E) cumulative data of the intensity of Gal-9 expression in adult male and female mice. (F) Representative gating strategy for the ImageStream analysis. Each dot represents data from an animal, mean ± SEM from multiple independent experiments. Fluorescence minus one (FMO), the mean fluorescence intensity (MFI). [file DataSheet_1.pdf]
